# Literary Notes

**Published:** 1897-08

**Authors:** 


### Literary Notes.

THE *Medical Record*, according to its annual custom, devoted its issue of June 12, 1897, to a consideration of summer health resorts, publishing a special article on that subject, ten pages and a half long, besides innumerable advertisements. This was a large and handsome number of this always enterprising magazine.

THE Buffalo State Hospital has lately issued its twenty-sixth annual report, which covers the year ending September 30, 1896. During this period there has been an increase in the population of the institution of 53 men and 210 women, total 263. Of this number, however, 207 were transfers of chronic cases from other institutions. The hospital accommodations have been enlarged during the year and its affairs are in a progressive and satisfactory state.

THE Manhattan Eye and Ear Hospital reports, Volume IV., January 1897, is an interesting brochure of about 100 pages, containing twenty papers by leading ophthalmologists, otologists and laryngologists, among whom are Drs. D. B. St. John Roosa, David Webster, Frank Van Fleet, M. D. Lederman and Charles H. Knight. It is printed by the Knickerbocker press (G. P. Putnam's Sons,) and it is needless to add that the mechanical execution is of the best.

THE *New Orleans Medical and Surgical Journal* celebrated its fiftieth anniversary by issuing a souvenir number for July, 1897. This journal was established in 1844, but suspended publication during the civil war, hence its fiftieth volume began with its jubilee number as indicated.

It is a magazine that always has been conducted with dignity and honor, never lowering the standard of medical propriety, and always contributing something toward the advancement of medical science. Its present editors point with just pride to this circumstance and they are entitled to receive the congratulations of their contemporaries throughout the United States on this honorable record. We observe with pleasure that this veteran magazine will prove its youthful vigor by revising its orthography, eliminating diphthongs wherever practicable, and otherwise place itself in the front rank of modern medical magazines. Our best wishes go out to our New Orleans contemporary for its continued success as a representative magazine.

THE first report of the board of managers of the Second Hospital for the Insane of the State of Maryland, gives a history of the establishment of what promises to be the most famous institution of its kind in the country. It has been established at "Springfield," near Sykesville, the old Patterson homestead, and it was from this mansion that Elizabeth Patterson, according to tradition, made her escape at night and rode on horseback to Baltimore, accompanied only by her negro groom, where she met Jerome Bonaparte at the famous ball and whom she soon afterward married.

It is an ideal location for a hospital; there is a succession of hills upon which to erect the buildings, giving natural drainage and the purest air, and the superintendent, Dr. George H. Rohé, has the knowledge and experience to put all these natural advantages to the best possible use in the construction and maintenance of a great state hospital for the insane.

---

THE *Indiana Medical Journal*, one of the best of our contemporaries, is always a welcome visitor. We often quote it and find it generally so sound on all important questions that we are pained to feel impelled to utter a word of criticism. We are surprised to find in its last issue an editorial that speaks in commendation of "yellow medical journalism." If there is anything that should bring the profession of medical journalism to blush and cause the American Medical Editors' Association to weep, it is that kind of periodic literature, to the consideration of which our Indiana contemporary devotes an editorial page, and which will be regarded in the light of a "boom." We do not believe that such was the intent; nevertheless, it will assuredly become so in effect.

---

THE *American X-ray Journal* made its appearance in May, 1897. Dr. Heber Robarts is the editor, and it hails from St. Louis. We fail to discover a substantial *raison d'etre* for this special line of journalism.

---

THE *Medical Register* is the name of a new journal that appeared in May, 1897. It is issued monthly under the auspices of the faculty and society of alumni of the Medical College of Virginia. It is edited by E. C. Levy, M. D., and Drs. John M. Upshur and Lewis C. Bosher constitute the advisory committee from the faculty. The office of publication is 826 East Main street, Richmond, Va.

W. B. Saunders, Esq., medical publisher, 925 Walnut street, Philadelphia, announces for publication during autumn and early winter the following book-list: An American text-book of genito-urinary and skin diseases, edited by L. Bolton Bangs, M. D., late professor of genito-urinary and venereal diseases, New York Post-Graduate Medical School and Hospital, and William A. Hardaway, M. D., professor of diseases of the skin, Missouri Medical College. An American text-book of diseases of the eye, ear, nose and throat, edited by G. E. deSchweinitz, M. D., professor of ophthalmology in the Jefferson Medical College, and B. Alexander Randall, M. D., professor of diseases of the ear in the University of Pennsylvania and in the Philadelphia Polyclinic. Macdonald's surgical diagnosis and treatment, by J. W. Macdonald, M. D., graduate of medicine of the University of Edinburgh; licentiate of the Royal College of Surgeons, Edinburgh; professor of the practice of surgery and of clinical surgery, Minneapolis College of Physicians and Surgeons. Anders' theory and practice of medicine, by James M. Anders, M. D., Ph. D., LL. D., professor of the theory and practice of medicine and of clinical medicine, Medico-Chirurgical College, Philadelphia. Senn's genito-urinary tuberculosis, by Nicholas Senn, M. D. Ph. D., LL. D., professor of the practice of surgery and of clinical surgery, Rush Medical College, Chicago. Penrose's gynecology, by Charles B. Penrose, M. D., professor of gynecology, University of Pennsylvania. Hirst's obstetrics, by Barton Cooke Hirst, M. D., professor of obstetrics, University of Pennsylvania. Moore's orthopedic surgery, by James E. Moore, M. D., professor of orthopedics and adjunct professor of clinical surgery, University of Minnesota, College of Medicine and Surgery. Heisler's embryology, by John C. Heisler, M. D., prosector to the professor of anatomy, Medical Department of the University of Pennsylvania. Mallory and Wright's pathological technique, by Frank B. Mallory, A. M., M. D., assistant professor of pathology, Harvard Medical School; assistant pathologist to the Boston City Hospital; and James H. Wright, A. M., M. D., instructor in pathology, Harvard Medical School; pathologist to the Massachusetts General Hospital.

New volume in Saunders' aid series: Sutton and Giles' diseases of women, by J. Bland Sutton, F. R. C. S., assistant surgeon to Middlesex Hospital, and surgeon to Chelsea Hospital, London; and Arthur E. Giles, M. D., B. Sc., Lond., F. R. C. S., Edin., assistant surgeon, Chelsea Hospital, London.

The *Columbus Medical Journal* devoted its issue of April 27, 1897, to the Association of Military Surgeons, that held its annual meeting at Columbus during the month of May. It is as handsome a specimen of medical journalism as we have seen in many a day. It is printed on fine book-paper and contains 42 engravings, for the most part half-tones of officers of the association and scenes in and about Columbus.

---

The *Northern Druggist* is the new name of what was known as the *Buffalo Druggist.* We fail to understand or appreciate the propriety of this change in name, for we think Buffalo is a pretty good first name for a magazine or newspaper, medical or lay. However, if our friends of the *Druggist* are satisfied we need not complain.

---

The *Pittsburg Medical Review*, that sterling representative of honorable medicine, has been transformed into the official organ of the medical society of the State of Pennsylvania and rechristened the *Pennsylvania Medical Journal.* The *Review* was too good a magazine to become an "organ," but the Pennsylvania society could not have selected a more worthy mouth-piece.

